# Evaluation on the Photosensitivity of 2,2′-Azobis(2,4-Dimethyl)Valeronitrile with UV

**DOI:** 10.3390/molecules22122219

**Published:** 2017-12-14

**Authors:** Yi Yang, Yun-Ting Tsai

**Affiliations:** 1 Shaanxi Key Laboratory of Prevention and Control of Coal Fire, School of Safety Science and Engineering, Xi’an University of Science and Technology, Xi’an 710054, China; yangyi_610@163.com; 2 School of Chemical Engineering and Technology, Xi’an Jiaotong University, Xi’an 710049, China

**Keywords:** azo compound, free initiator, calorimetric and product analysis technology, kinetic parameters, environmental protection

## Abstract

Azo compounds have high exothermic characteristics and low thermal stability, which have caused many serious thermal accidents around the world. In general, different locations (e.g., equatorial or polar regions) have different UV intensities. If the azo compound exists in an inappropriately stored or transported condition, the decrease in thermal stability may cause a thermal hazard or ageing. 2,2′-Azobis(2,4-dimethyl)valeronitrile (ADVN) is investigated with respect to the thermal stability affected by UV exposure at 0, 6, 12, and 24 h. When ADVN is exposed to 24 h of UV (100 mW/m^2^ and 254 nm), *T*_0_ is not only advanced, but the mass loss is also increased during the main decomposition stage. In addition, the apparent activation energy and integral procedural decomposition temperature (*IPDT*) of ADVN exposed to 24 h of UV is calculated by kinetic models. Therefore, the prevention mechanism, thermal characteristics, and kinetic parameters are established in our study. We should isolate UV contacting ADVN under any situations, avoiding ADVN being aged or leading to thermal runaway. This study provided significant information for a safer process under changing UV exposure times for ADVN. Furthermore, the research method may serve as an important benchmark for handling potentially hazardous chemicals, such as azo compounds described herein.

## 1. Introduction

In view of loss prevention, the appraisal of thermal characteristics for reactive chemicals has been a major concern of chemical industries. The main reason for many accidents is insufficient knowledge of reactants, intermediates, products, or various catalysts. In general, a small change of temperature or pressure may sporadically cause a serious runaway reaction for highly-energetic chemicals. The most hazardous information can be obtained from the literature, but if this information is scanty, the related safety parameters should be assessed by proper thermal analysis technology [1,2,3].

**Figure 1:**
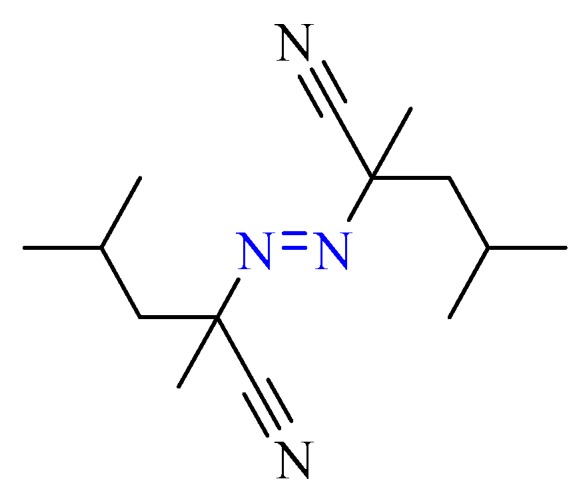
Chemical structure of ADVN.

**Figure 2:**
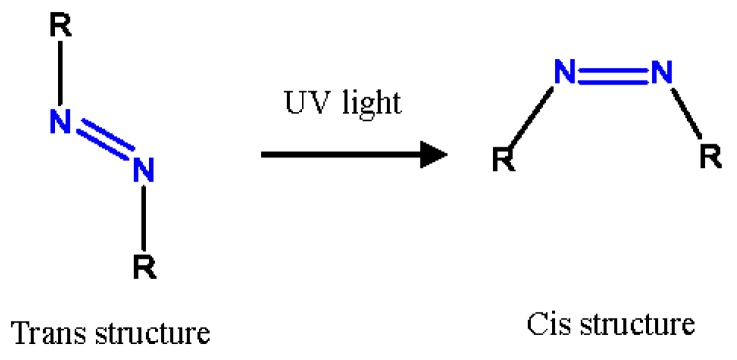
Light effect for the trans-cis isomerization mechanism of ADVN.

2,2′-Azobis(2,4-dimethyl)valeronitrile (ADVN) (the chemical structure is shown in Figure 1), a common azo compound, is an excellent free radical supplier for chemical processes. When azo is involved in an exothermic reaction, it can amply provide free radicals and energy for synthesis of organic compounds or polymers, such as styrene, methyl acrylate, epoxy resin, or propylene, as an initiator, cross-linking, or curing agent. However, ADVN has thermal instability and sensitivity because of its –N=N– structure, which may incur violent thermal decomposition by external fire, other igniting sources, or irradiation [4,5,6,7,8]. In the past, many thermal accidents have occurred because the inherent safety of chemicals is not explicit and the accident process is unexpected, which may result in the stored temperature, cooling system, or pressure relief system to be wrongly designed for safety considerations. Wanasekara et al. in 2011 indicated that the increase in the temperature and exposure time may lead to surface degradation and embrittlement of fibers. Wang et al. in 2008 mentioned that the UV light can cause the trans-cis isomerization mechanism of azo compounds (see Figure 2). Wang and Wang in 2008 also indicated that the azo with a trans isomer has high thermal stability and low energy intensity [9,10,11]. Thus, it is crucial that we understand the thermal hazard behaviors for azo which can be used efficiently and safely during operating, transportation, and storage [12]. In the past, most of the research of ADVN for runaway excursion has been the analysis of pure substances. However, the photo effect of ADVN has rarely been explored. Since ADVN has extreme photosensitivity with UV, the decomposition mechanism may be changed with the exposure time and mass [13]. Often, UV irradiation exists ubiquitously, and even its intensity may be different between location latitudes. Simultaneously, if the optical isolation of UV for the package or container is not complete, the photo effect may decrease the thermal stability of ADVN, resulting in the increased risk of a thermal runaway reaction [14].

The calorimetry and kinetic models are used to analyze the thermal stability parameters, such as apparent onset temperature (*T*_0_), peak temperature (*T*_p_), final temperature (*T*_f_), apparent activation energy (*E*_a_), frequency factor (ln*k*_0_), and reaction order (*n*), to establish the hazard characteristics and to explore the decomposition products by employing differential scanning calorimetry (DSC) and thermogravimetry (TG) for determination of UV effects on ADVN [15,16].

## 2. Results and Discussion

### 2.1. TG Results

**Figure 3:**
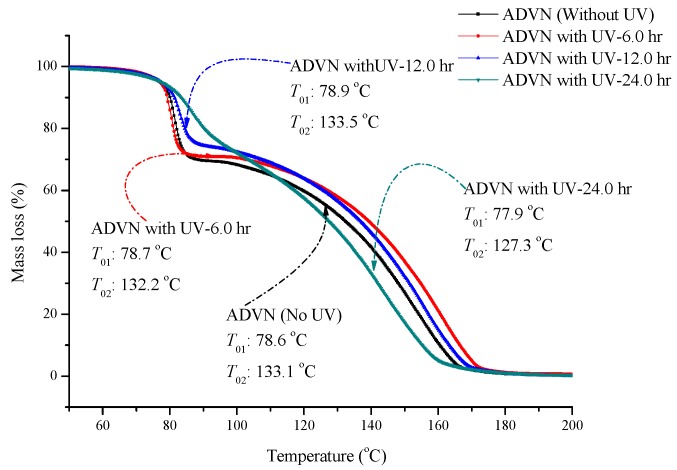
TG thermal curve of ADVN and ADVN with different exposure durations of UV/6, 12 and 24 h.

Figure 3 shows the mass loss versus temperature diagram from TG testing for ADVN and ADVN with different exposure durations of 6, 12 and 24 h of UV irradiation at a heating rate 20 °C/min. There are two decomposition stages for ADVN and ADVN exposed to UV, and the main decomposition stage is the second stage. We found that when ADVN was exposed to 6 and 12 h of UV, the profiles of the TG curve are not obviously different between each other, and for both *T*_0_ is 78 °C. However, although the *T*_0_ of the first stage of ADVN is close to ADVN exposed to 24 h of UV, the *T*_0_ of the second decomposition stage is curtailed from 132 °C to 127 °C and the mass loss is increased from 70% to 80%. According to the literature [17], *T*_0_ and mass loss can be used to determine the thermal stability for chemicals. Therefore, if ADVN is exposed to a UV intensity of 100 mW/m^2^ for over 24 h, the onset temperature and temperature of the maximum mass loss can decrease, causing abnormal aging or lessening the thermal stability during preparation, storage, transportation, and operating conditions.

**Figure 4:**
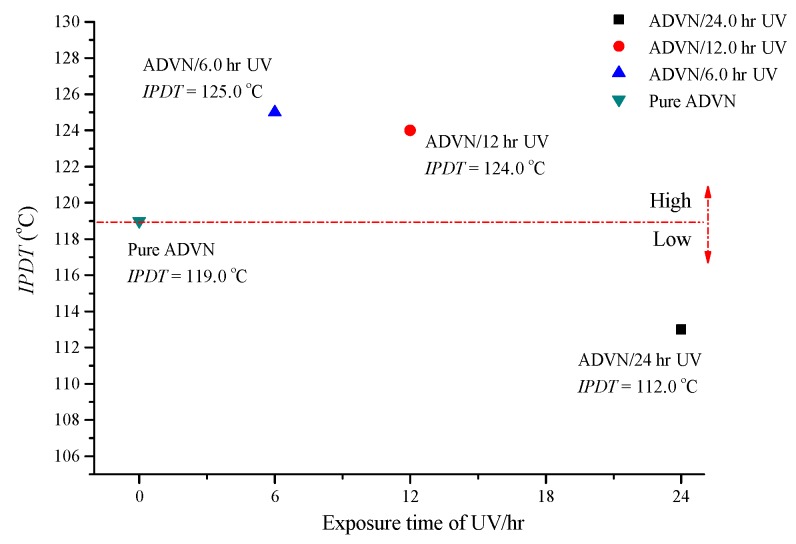
Calculation of *IPDT* for ADVN with different exposure durations of UV/6, 12, and 24 h by TG testing.

In Figure 4, we compared the *IPDT* for ADVN and ADVN with different exposure durations of 6, 12 and 24 h of UV irradiation, corresponding to values of 119, 125, 124 and 112, respectively. The results showed that when the exposure time of UV is 6 and 12 h, the *IPDT* could be increased, but the *IPDT* of ADVN is decreased after exposure to UV for 24 h. According to the literature, the thermal characteristics of chemicals are determined by energy and states of chemical bonds [18]. UV light can lead to the formation of the trans-cis isomerization of ADVN, causing a decrease in the thermal stability. In general, 24 h is usually considered as a standard emergent time or transportation for chemical industries, so that ADVN should avoid being in contact with UV irradiation under any conditions. The TG and *IPDT* results for ADVN exposed to 6, 12, and 24 h of UV at a heating rate 20 °C are listed in Table 1.

**Figure 5:**
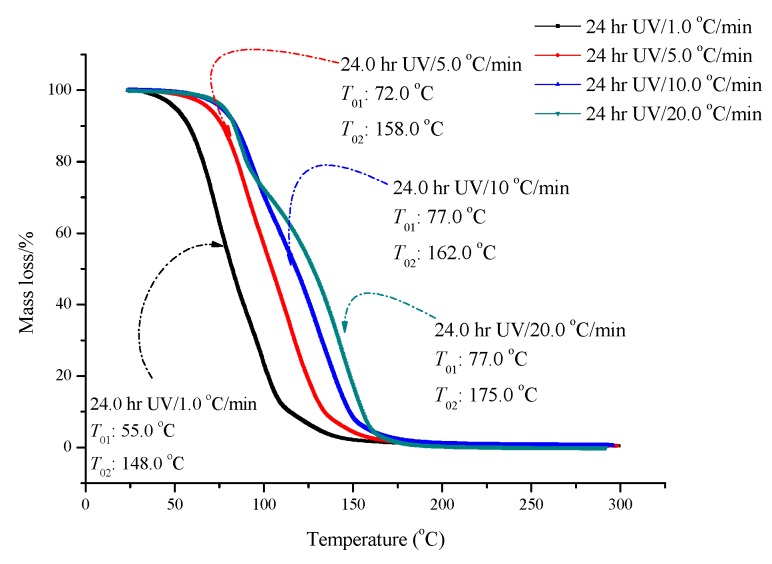
TG thermal curve of ADVN with UV/24 h at heating rates of 1, 5, 10, and 20 °C/min.

**Figure 6:**
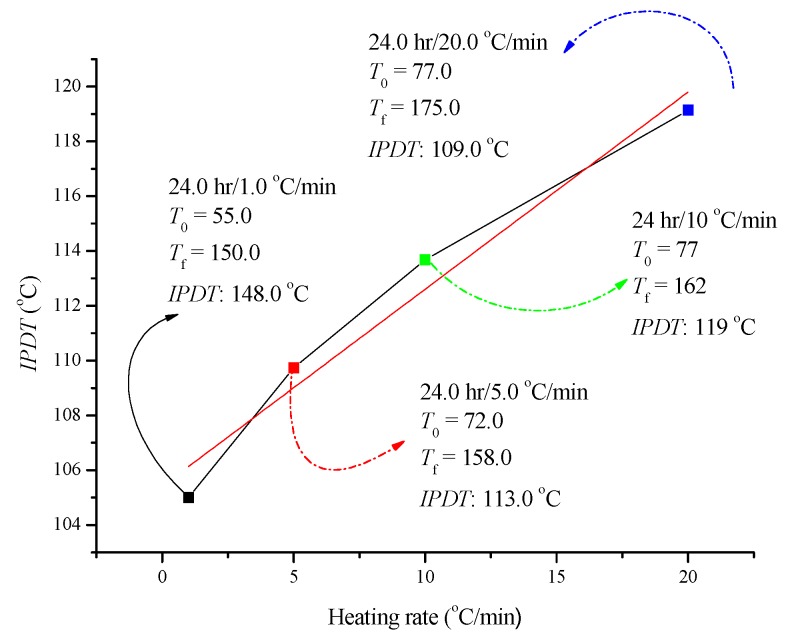
Linear dependence of *IPDT* for ADVN at 24 h UV at heating rates of 1, 5, 10, and 20 °C/min.

Based on the above-mentioned results, the following experiments focused on the UV effect for ADVN with an exposure time of 24 h. ADVN exposed to UV for 24 h was tested by TG at heating rates of 1, 5, 15 and 20 °C, as delineated in Figure 5. We also compared the *IPDT* for the results of four heating rates and the value of *IPDT* was increased from 101 °C to 119 °C with an increasing heating rate. Moreover, we plotted the *IPDT* versus the heating rate curve, and there was an extremely high *R*^2^ value from the linear regression. According to the results, the heating rate can determine the *IPDT* for the decomposition reaction of ADVN exposed to UV for 24 h. From the viewpoint of safer process design, when the operating conditions of a chemical process involving ADVN exposed to UV for 24 h need a low reaction temperature, a low heating rate should be proper and reliable. However, a high heating rate is better for a high reaction temperature. *IPDT* will be used as a basic, rudimentary, and dependable thermal stability parameter for future study. The linear dependence of *IPDT* with different heating rates is depicted in Figure 6.

### 2.2. Determination of Kinetic Models for ADVN With UV Irradiation

**Figure 7:**
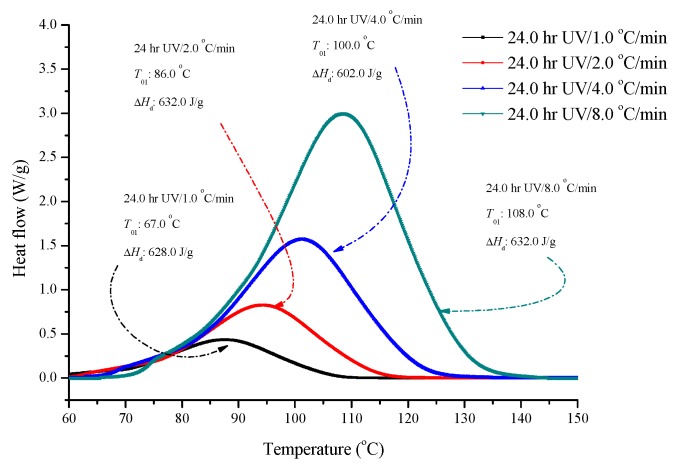
DSC thermal curves of ADVN with 24 h UV at heating rates of 1, 2, 4 and 8 °C/min.

**Figure 8:**
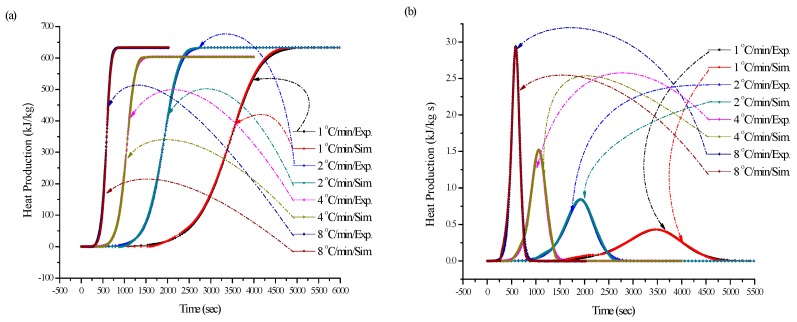
(**a**) Heat production versus time and (**b**) heat production rate versus time of model fitting with non-isothermal DSC at heating rates of 1, 2, 4, and 8 °C/min for ADVN with 24 h UV via experiments and simulations.

To investigate the reaction mechanism and predict the kinetic behaviors for ADVN exposed to 24 h of UV, non-isothermal kinetic analysis was conducted based on DSC data, which is illustrated in Figure 7. Followed ICTAC recommendations, the kinetic calculation requests three up temperature programs. The complex reaction model can use the non-linear model-fitting method to test the kinetic parameters [19]. Figure 8 displays (a) heat production versus time and (b) heat production rate versus time by experiments and simulations. From the simulation results, we observed that there was significant curve fitting and nearly the same kinetic parameters, including *n*, *E*_a_, *A*, and Δ*H*_d_, for both plots of heat production and heat production rate versus time of ADVN exposed to 24 h of UV at heating rates of 1, 2, 4 and 8 °C/min by simple *n*th order kinetic models. Therefore, ADVN exposed to 24 h of UV is determined as an *n*th order reaction, instead of autocatalysis, and the obtained kinetic parameters can be used to predict other heating rates or reaction temperatures for reliable information in the future studies. The related kinetic parameters are given in Table 2 [20].

## 3. Materials and Methods

### 3.1. Sample

The sample chosen was ADVN 98 wt %, which was purchased from ACE Chemical Corp., Taoyuan, Taiwan. ADVN is light sensitive and thermally unstable, so it should be maintained in a dark space and at a low temperature of 4 °C. The UV instrument was purchased from Uvitron International, Inc. (West Springfield, MA, USA). The exposure time and intensity of UV irradiation is 6, 12 and 24 h, respectively, to determine the UV effect on ADVN. The intensities/irradiance of their UV light exposures is 100 W/m^2^ and 254 nm. This value is the average intensity of UV at noon during the summer in China.

### 3.2. Differential Scanning Calorimetry (DSC)

Temperature-controlled thermal curves of DSC experiments facilitate understanding the exothermic or endothermic reaction of a chemical, such as crystallization, curing reaction, phase change, or thermal decomposition reaction. Thus, it can be used as a safety assessment methodology to provide the thermal hazard information for ADVN. The type of DSC is selected in the Mettler DSC 821^e^. To investigate thermal characteristics of ADVN with UV irradiation, we heated it from 30 °C to 300 °C at different heating rates, here, 1, 2, 4 and 8 °C/min, using the DSC test. The sample amount for each experiment was approximately 1.5–5 mg and the sample was sealed in a gold-plated crucible [21].

### 3.3. Thermogravimetry (TG)

Thermogravimetry, using a PerkinElmer Clarus 680 unit, was used to analyze the thermal decomposition products for ADVN and ADVN exposed to UV with different times of 6, 12 and 24 h. The TG experiments were performed from ambient temperature to 300 °C with heating rates of 1, 5, 10 and 20 °C/min in nitrogen gas purged at a flow rate of 100 mL/min [22].

### 3.4. Evaluation of Integral Procedural Decomposition Temperature (IPDT)

An evaluation parameter of thermal stability, *IPDT*, was used to consider and explore the overall thermal stability during the decomposition process. Since thermal stability involves three crucial factors of chemicals, including initial reaction, end reaction, and ratio for mass loss, *IPDT* was created based on different thermogravimetric regions of the TG curves. The equations of *IPDT* are as follows [23,24]:

(1) $$IPDT=A{}^{\circ}\times K{}^{\circ}\times \left(T_{f}-T_{i}\right)+T_{i}$$

**Figure 9:**
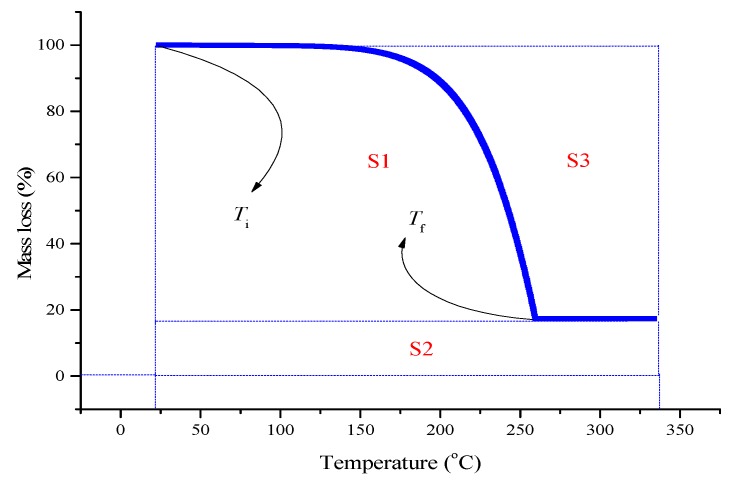
Schematic diagram for the conception of *IPDT* [23,24].

(2) A°=(S1+S2)/(S1+S2+S3)

(3) K°=(S1+S2)/(S1)

where *A*° and *K*° is the proportion of mass loss. *T*_i_ and *T*_f_ are the mass loss at the initial temperature and final temperature, respectively. S1, S2, and S3 are different partitioned regions. S1 is the thermogravimetric area under the TG curve; S2 is the thermogravimetric area of the non-mass loss; and S3 is the thermogravimetric area above the TG curve. The schematic diagram of *IPDT* is shown in Figure 9.

### 3.5. Non-Linear Regression Method to Decide the Reaction Kinetics of ADVN/UV Irradiation

In general, typical fundamental reactions of azo are *n*th order and autocatalytic; they may be composed of either a one-stage or a multi-stage reaction. However, linear regression methods, such as Kissinger, Ozawa, or Friedman models, are unable to predict complex chemical reactions of azo. Therefore, the non-linear regression method is usually used to directly fit the thermal curve of chemicals through kinetic models to appraise the chemical reaction stages and various kinetic parameters, such as *n*, *E*_a_, *A*, and the autocatalytic constant (*z*) for use in chemical engineering processes. The *n*th order and autocatalytic reaction models are presented in Equations (4) and (5), correspondingly [25,26,27,28,29]:

(4) $$\frac{d\alpha }{dt}=A\exp\left(\frac{-E_{a}}{RT}\right){\left(1-\alpha \right)}^{n}$$

(5) $$\frac{d\alpha }{dt}=A\exp\left(\frac{-E_{a}}{RT}\right){\left(1-\alpha \right)}^{n_{1}}(\alpha^{n_{2}}+z)$$

where *n*_1_ and *n*_2_ are reaction orders for the two different reaction stages, *α* is the degree of conversion, and *z* is the autocatalytic constant.

## 4. Conclusions

We tested the thermal stability of ADVN exposed to UV at 100 W/m^2^ and 254 nm, which is the average intensity of UV at noon during the summer in China. Based on the experimental results, when ADVN is exposed for 6 h and 12 h, the *IPDT* is decreased, demonstrating that the short exposure time cannot affect the thermal stability for ADVN. However, when ADVN is exposed to 24 h of UV, *T*_0_ is not only advanced, but the mass loss can also increase during the main decomposition stage. UV light can cause ADVN to the trans-cis isomerization, causing the decrease in the thermal stability. We should isolate UV contacting ADVN under any situation, avoiding ADVN being aged or leading to thermal runaway. In addition, *IPDT* and non-isothermal kinetic models are carried out to evaluate the thermal stability, reaction mechanism, and related kinetic parameters of ADVN exposed to UV irradiation. From the simulation results, we observed that there was significant curve fitting and nearly the same kinetic parameters. ADVN exposed to 24 h of UV is determined as an *n*th order reaction, instead of autocatalysis, and the obtained kinetic parameters can be used to predict other heating rates or reaction temperatures for reliable information in future studies. This study provided significant information on a safer process under changing exposure time of UV for ADVN. Furthermore, the research method may serve as an important benchmark for handling potentially hazardous chemicals, such as azo compounds described herein.

## Figures and Tables

**Table 1:** Thermal stability parameters by TG testing for ADVN and ADVN exposed to 6, 12 and 24 h of UV at a heating rate 20 °C.

Sample	*T*_01_ (°C)	*T*_02_ (°C)	Mass Loss (First Stage) (%)	Mass Loss (Second Stage) (%)	*IPDT*/°C
ADVN/without UV	78.6	132.1	30.0	70.0	119.0
ADVN/6 h of UV	78.7	133.2	30.0	70.0	125.0
ADVN/12 h of UV	78.9	133.5	25.0	75.0	124
ADVN/24 h of UV	77.9	127.3	20.0	80.0	112.0

**Table 2:** Reaction kinetic simulation for ADVN with UV/24 h using non-linear regression methods at heating rates of 1, 2, 4, and 8 °C/min.

Heating Rate (°C/min)	*E*_a_ (kJ/mol)	ln (*A*) (1/s)	Reaction Order (*n*)	Δ*H*_d_ (kJ/kg)
1.0	137.0	39.0	1.4	632.0
2.0	144.0	42.0	1.5	632.0
4.0	140.0	40.0	1.4	603.0
8.0	138.0	39.0	1.5	633.0
